# Taking Education to the World: Implementing Aging Curriculum in Faith Communities

**DOI:** 10.1093/geroni/igaa057.1746

**Published:** 2020-12-16

**Authors:** Lauren Bouchard

**Affiliations:** Concordia University Chicago, Chicago, Illinois, United States

## Abstract

Religious communities are uniquely able to serve the needs of older adults, yet very little aging related training exists for lay people and clergy within religious congregations. This talk will discuss the needs of older adults in faith communities, intergenerational interventions, and formal educational training programs such as the Specialist in Aging Ministries program (SAM) developed by Concordia University Chicago as a way to educate and support caregivers and older adults in real world settings. The speaker will also discuss the ways gerontology curriculum has personally impacted her applied work with elders in faith communities including next steps for building and implementing curriculum and training for caregivers, older adults, and the community at large. Sociocultural implications and future directions will also be discussed.

